# Hypercholesterolemia Accelerates the Aging Phenotypes of Hematopoietic Stem Cells by a Tet1-Dependent Pathway

**DOI:** 10.1038/s41598-020-60403-w

**Published:** 2020-02-27

**Authors:** Guodong Tie, Jinglian Yan, Lyne Khair, Amanda Tutto, Louis M. Messina

**Affiliations:** 1 Division of Vascular and Endovascular Surgery, University of Massachusetts Medical School, Worcester, MA 01655 USA; 2 Diabetes Center of Excellence and Division of Vascular and Endovascular Surgery, University of Massachusetts Medical School, Worcester, MA 01655 USA

**Keywords:** Haematopoietic stem cells, Risk factors

## Abstract

Hypercholesterolemia accelerates the phenotypes of aging in hematopoietic stem cells (HSCs). As yet, little is known about the underlying mechanism. We found that hypercholesterolemia downregulates Ten eleven translocation 1 (*Tet1*) in HSCs. The total HSC population was increased, while the long-term (LT) population, side population and reconstitution capacity of HSCs were significantly decreased in Tet1^−/−^ mice. Expression of the Tet1 catalytic domain in HSCs effectively restored the LT population and reconstitution capacity of HSCs isolated from Tet1^−/−^ mice. While Tet1 deficiency upregulated the expression of p19 and p21 in HSCs by decreasing the H3K27me3 modification, the restoration of Tet1 activity reduced the expression of p19, p21 and p27 by restoring the H3K27me3 and H3K36me3 modifications on these genes. These results indicate that Tet1 plays a critical role in maintaining the quiescence and reconstitution capacity of HSCs and that hypercholesterolemia accelerates HSC aging phenotypes by decreasing Tet1 expression in HSCs.

## Introduction

Adult tissue-specific stem cells maintain tissue homeostasis and regenerative potential. In the bone marrow, normal hematopoiesis relies on hematopoietic stem cells (HSCs) that differentiate through a number of committed progenitors and give rise to mature blood cells^1^. To maintain their capacity for normal hematopoiesis and to protect the HSC compartment from exhaustion, the majority of HSCs remain quiescent and only a few of them enter the cell cycle^2,3^.

Aging is known to have dramatic effects on the function of HSCs. In murine models, serially transplanted HSCs from aged mice are less functional than their younger counterparts and eventually become exhausted^4,5^. The aging of HSCs causes an expansion of the HSC compartment, a decreased repopulation capacity, reduced lifespan and skewed myeloid differentiation potential^6^. The molecular mechanisms responsible for HSC aging is complicated. A number of mechanistic cues have been reported. These include oxidant stress^7^, metabolic stress^8^, telomere erosion^9^, deregulation of lineage specification^10^, loss of cell polarity^11^, activation of inflammatory signaling cascades^12^ and DNA damage accumulation^13^. However, these mechanisms do not explain all of the HSC functional defects induced by aging, suggesting that other regulatory pathways must participate in this process of HSC aging.

Recent studies indicate that epigenetic regulation, including DNA methylation, histone modification and chromatin structure, plays important roles in the physiology and pathology of hematopoiesis. Methylation at carbon atom 5 of cytosine (5-mC) is the predominant repressive mark of DNA epigenetic modifications. The ten-eleven translocation (Tet) family, including Tet1, Tet2 and Tet3, oxidizes 5-mC and initiates the demethylation of DNA^14–16^. Tet1 and Tet2 have been shown to regulate the self-renewal, proliferation and differentiation of HSCs^14,17,18^. The expression of Tet1 is significantly decreased in HSCs of aged mice, suggesting that Tet1 participates in the regulation of HSC aging phenotypes^19^. However, the underlying mechanisms are as yet unknown.

Obesity and its co-morbidities have been closely associated with a functional decline in the HSC compartment^20–23^. In our previous study, we showed that hypercholesterolemia, a major co-morbidity of obesity, caused a significant reduction in long-term HSC populations, telomere length, and reconstitution capacity of HSCs^24^. Several key cell cycle regulators including p19, p27 and p21 were upregulated in HSCs from hypercholesterolemic mice. Together with previous reports^25,26^, these findings indicate that hypercholesterolemia accelerates the phenotypes of aging in HSCs. Our recent study also showed that *Tet1* was specifically decreased in HSCs isolated from hypercholesterolemic mice. With these findings, we hypothesize that the decrease in Tet1 expression alters the epigenetic regulation and accelerates the aging phenotypes of HSCs in hypercholesterolemic mice.

## Results

### The expression of *Tet1* is downregulated in HSCs isolated from hypercholesterolemic mice

**Figure 1:**
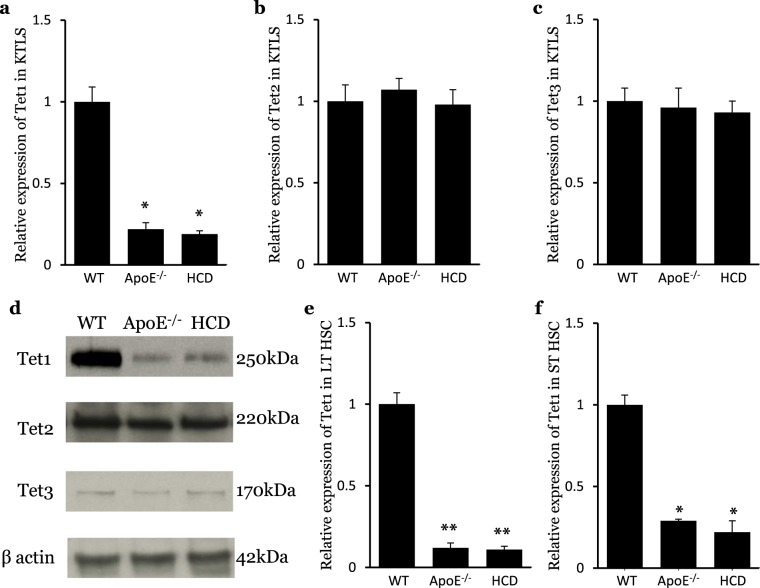
The expression of *Tet1* is downregulated in HSCs isolated from hypercholesterolemic mice. (**a**) Relative expression of *Tet1* in HSCs from WT, ApoE^−/−^ and HCD mice measured by RT-PCR. **(b)** Relative expression of *Tet2* in HSCs from WT, ApoE^−/−^ and HCD mice measured by RT-PCR. **(c)** Relative expression of *Tet3* in HSCs from WT, ApoE^−/−^ and HCD mice measured by RT-PCR. (n = 5, *p < 0.05, vs WT). **(d)** The expression of Tet1, Tet2 and Tet3 in HSCs, measured by Western blot. **(e)** Expression of *Tet1* in LT HSCs from WT, ApoE^−/−^ and HCD mice measured by RT-PCR. **(f)** Expression of *Tet1* in ST HSCs from WT, ApoE^−/−^ and HCD mice measured by RT-PCR. (n = 5, *p < 0.05; **p < 0.01; vs WT).

The enzymes of the Tet family, especially Tet1 and Tet2, are highly expressed in mouse HSCs^27,28^. In this study, we found that the expression of *Tet1* was significantly lower in HSCs (KTLS cells, defined as Sca1^+^cKit^+^Lin^−^CD90.1^−/low^) isolated from hypercholesterolemic mice than in that of wild type (WT) control mice (Fig. 1a). No difference was found in the expression of Tet2 or Tet3 (Fig. 1b,c). The expression of Tet1, 2 and 3 was confirmed at the protein level by Western blot (Fig. 1d). The expression of *Tet1* was significantly decreased in both long-term (LT) HSCs (defined as CD34^−^Flk2^−^ KTLS cells) and short-term (ST) HSCs (Fig. 1e,f). These results are consistent with our previous observations^17^.

### Tet1 deficiency induces loss of quiescence and reduces the long-term population of HSCs

**Figure 2:**
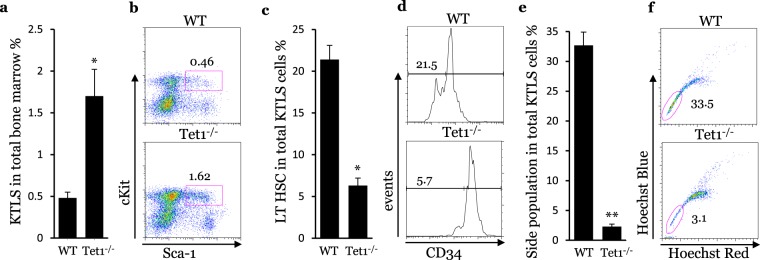
Tet1 deficiency causes expansion of the HSC compartment, and decreases long-term populations and side populations of the HSC compartment. (**a)** KTLS cells in bone marrow of WT and Tet1^−/−^ mice. **(b)** Representative FACS dot plot. **(c)** Long-term populations in HSC compartment of WT and Tet1^−/−^ mice. **(d)** Representative FACS histogram. **(e)** Side populations in HSC compartment of WT and Tet1^−/−^ mice. **(f)** Representative FACS dot plot. (n = 5, *p < 0.05; **p < 0.01; vs WT).

To analyze the role of Tet1 in the HSC compartment, we generated Tet1^−/−^ mice. The frequency of HSCs in the bone marrow of Tet1^−/−^ mice (1.62 ± 0.34%) was significantly greater than the frequency observed in the bone marrow of WT mice (0.46 ± 0.09%) (Fig. 2a,b). However, the long-term population of HSCs in Tet1^−/−^ mice (5.7 ± 0.9%) was significantly lower in WT mice (21.5 ± 2.4%) (Fig. 2c,d), which is in agreement with a previous study^28^. Similarly, the quiescent side-population of HSCs was reduced in Tet1^−/−^ mice (Fig. 2e,f). Furthermore, FACS analysis showed that the expression of Ki67, a marker of cell proliferation, was significantly higher in both LT and ST HSC populations in Tet1^−/−^ mice then in WT mice (Supplementary Fig. 1a,b). On the other hand, the expression of annexin V, a marker of cell apoptosis, was identical in HSC populations of Tet1^−/−^ and WT mice (Supplementary Fig. 1c,d). PCR analysis showed that Tet1 deficiency and hypercholesterolemia did not change the expression of HSC markers, including CD34, Sca-1 and cKit (Supplementary Fig. 2). These results indicate that Tet1 deficiency induces the loss of quiescence and reduces the LT population of HSCs.

We further analyzed the effects of Tet1 deficiency onto the downstream multipotent progenitors (MPPs) of HSCs. MPP2 was identified as Lin- Sca-1^+^ cKit^+^ Flk2^−^ CD150^+^ CD48^+^. MPP3 was identified as Lin^−^ Sca-1^+^ cKit^+^ Flk2^−^ CD150^−^ CD48^+^. MPP4 was identified as Lin^−^ Sca^−^1^+^ cKit^+^ Flk2^+^ CD150^−^. Lymphoid-primed MPP (LMPP) was identified as Lin^−^ Sca-1^+^ cKit^+^ Flk2^hi^ CD150^−^ CD34^+^ (Supplementary Fig. 3). The MPP3 and MPP4 compartments were significantly increased in Tet1^−/−^ mice, while the compartments of MPP2 and LMPP did not show any change (Supplementary Fig. 3a,b). Our previous studies had shown that Tet1 deficiency decreased their differentiation towards natural killer T cells and γδT cells^17^ and increased the differentiation towards pro-inflammatory monocytes and macrophages. These results indicate that Tet1 deficiency alters the differentiation of HSCs to multiple cell lineages.

The Tet1 expression in MPP2, MPP3 and MPP4 of ApoE^−/−^ mice was slightly lower than in WT mice, whereas the expression of Tet1 in LMPP was unchanged (Supplementary Fig. 4a). The expression of Tet2 and Tet3 did not change in any of the MPP compartments of ApoE^−/−^ mice (Supplementary Fig. 4b,c). These results indicate that the defects of Tet1 deficiency induced by hypercholesterolemia occur almost exclusively in the HSCs compartment rather than in downstream progenitor compartments.

### Tet1 deficiency reduces the reconstitution capacity and lifespan of HSCs and thereby accelerates their phenotypes of aging

**Figure 3:**
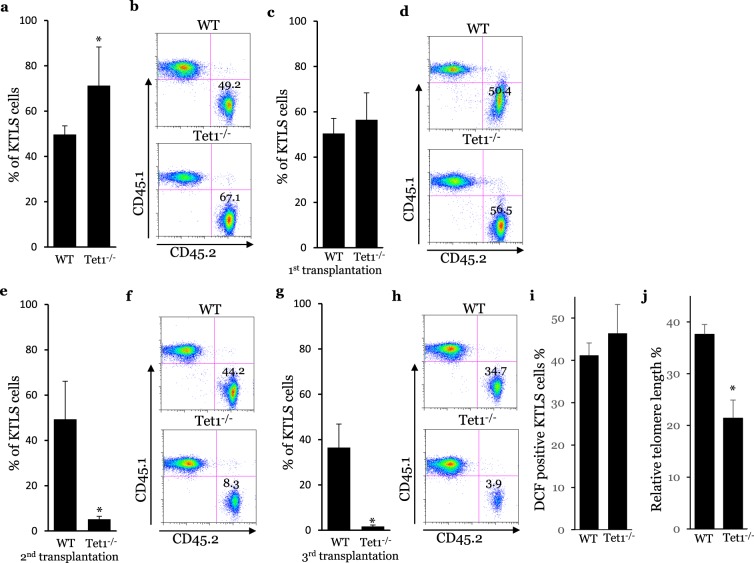
Tet1 deficiency reduces the life span and reconstitution capacity of HSCs. (**a)** Reconstitution capacity of LT HSCs of WT and Tet1^−/−^ mice. **(b)** Representative FACS dot plot. (n = 6, *p < 0.05, vs WT). **(c)** Reconstitution capacity of KTLS cells of WT and Tet1^−/−^ mice after the first transplantation. **(d)** Representative FACS dot plot. **(e)** Reconstitution capacity of KTLS cells of WT and Tet1^−/−^ mice after the second transplantation. **(f)** Representative FACS dot plot. (n = 6, *p < 0.05, vs WT). **(g)** Reconstitution capacity of KTLS cells of WT and Tet1^−/−^ mice after the third transplantation. **(h)** Representative FACS dot plot. **(i)** Oxidant levels in KTLS cells of WT and Tet1^−/−^ mice. **(j)** Telomere length in KTLS cells of WT and Tet1^−/−^ mice. (n = 6, *p < 0.05, vs WT).

A major manifestation of HSC aging is the decline of their reconstitution capacity and lifespan^29^. The LT HSC population plays an essential role in the reconstitution capacity of HSCs. In order to analyze repopulating capacity in our model, we isolated LT HSCs from Tet1^−/−^ and WT mice and measured their reconstitution capacity using a competitive transplantation assay. In agreement with their high proliferative phenotype, the LT HSCs from Tet1^−/−^ mice displayed greater reconstitution capacity (Fig. 3a,b), which was supported by a previous study^28^. However, the serial competitive transplantations showed that the KTLS cells (total HSCs) from Tet1^−/−^ mice, including LT and ST HSCs, gradually lost their reconstitution capacity after the second and third competitive transplantation (Fig. 3c–h). In our first transplantation, we found that Tet1^−/−^ KTLS cells contributed 56% of bone marrow reconstitution, while WT KTLS cells made the contribution around 50%. This difference is not statistically significant. Aifantis and his group used whole bone marrow in their primary transplantation and found that whole bone marrow cells from Tet1^−/−^ mice showed a higher reconstitution capacity^28^. The difference between our first transplantation and Aifantis’ primary transplantation might be due to the difference of transplanted cells. We used KTLS cells which were purified stem cell population and do not include CMPs, CLPs, MEPs, GMPs, MoPs and other hematopoietic progenitors, while Aifantis and his group used whole bone marrow cells^28^. Oxidant stress and telomere erosion have been identified as mechanistic cues in the process of HSC aging. Tet1 deficiency did not increase oxidant stress (Fig. 3i) but still induced significant telomere erosion in HSC compartment (Fig. 3j). These results indicate that Tet1 deficiency reduces the reconstitution capacity and lifespan of HSCs and thereby accelerates their phenotype of aging.

### Restoration of Tet1 activity eliminates the aging phenotype in HSCs

**Figure 4:**
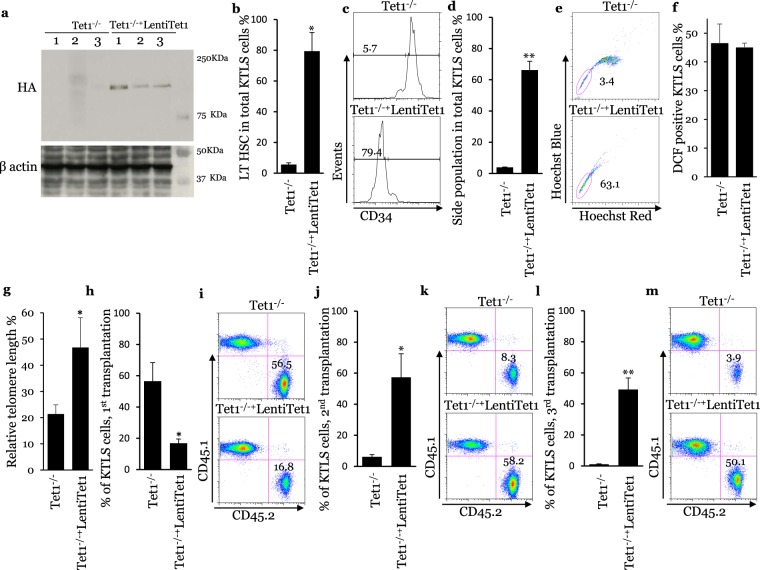
The expression of the Tet1 catalytic domain rescues quiescence, long-term population, life span and reconstitution capacity of HSCs isolated from Tet1^−/−^ mice. (**a)** Expression of Tet1 catalytic domain in HSCs. **(b)** Long-term populations in Tet1^−/−^ and Tet1^−/−^+LentiTet1 HSCs. **(c)** Representative FACS histogram. **(d)** Side populations in Tet1^−/−^ and Tet1^−/−^+LentiTet1 HSCs. **(e)** Representative FACS dot plot. (n = 6, *p < 0.05; **p < 0.01; vs Tet1^−/−^). (**f**) Oxidant levels of Tet1^−/−^ and Tet1^−/−^+LentiTet1 HSCs. (**g**) Telomere length of Tet1^−/−^ and Tet1^−/−^+LentiTet1 HSCs. (**h**) Reconstitution capacity of Tet1^−/−^ and Tet1^−/−^+LentiTet1 HSCs after the first transplantation. (**i**) Representative FACS dot plot. **(j**) Reconstitution capacity of Tet1^−/−^ and Tet1^−/−^+LentiTet1 HSCs after the second transplantation. (**k**) Representative FACS dot plot. (**l**) Reconstitution capacity of Tet1^−/−^ and Tet1^−/−^+LentiTet1 HSCs after the third transplantation. (**m**) Representative FACS dot plot. (n = 6, *p < 0.05; **p < 0.01; vs Tet1^−/−^).

We next asked if the restoration of Tet1 activity could rescue the LT population and reconstitution capacity of HSCs from Tet1^−/−^ mice. HSCs from Tet1^−/−^ mice were transfected with a lentiviral vector expressing the murine Tet1 catalytic domain. The expression was confirmed by Western blot (Fig. 4a). FACS analysis showed that the LT population in HSCs expressing the Tet1 catalytic domain was significantly larger than in control HSCs from Tet1^−/−^ mice (Fig. 4b,c). Similarly, the quiescent side population significantly increased in HSCs expressing the Tet1 catalytic domain (Fig. 4d,e). The restoration of Tet1 activity did not change oxidant levels (Fig. 4f) but rescued the telomere length (Fig. 4g). Next, we measured the reconstitution capacity of the Tet1^−/−^ HSCs that expressed the Tet1 catalytic domain. These cells displayed a more limited reconstitution capacity after the first transplantation than those of control HSCs from Tet1^−/−^ mice (Fig. 4h,i). However, they reconstituted 58.2 ± 11.3% and 50.1 ± 5.4% of HSCs in the bone marrow of recipient mice after the second and third transplantations, respectively, which was significantly greater than the reconstitution capacity of HSCs from Tet1^−/−^ mice (Fig. 4j–m).

### Hypercholesterolemia accelerates HSC aging phenotypes by decreasing Tet1 expression

**Figure 5:**
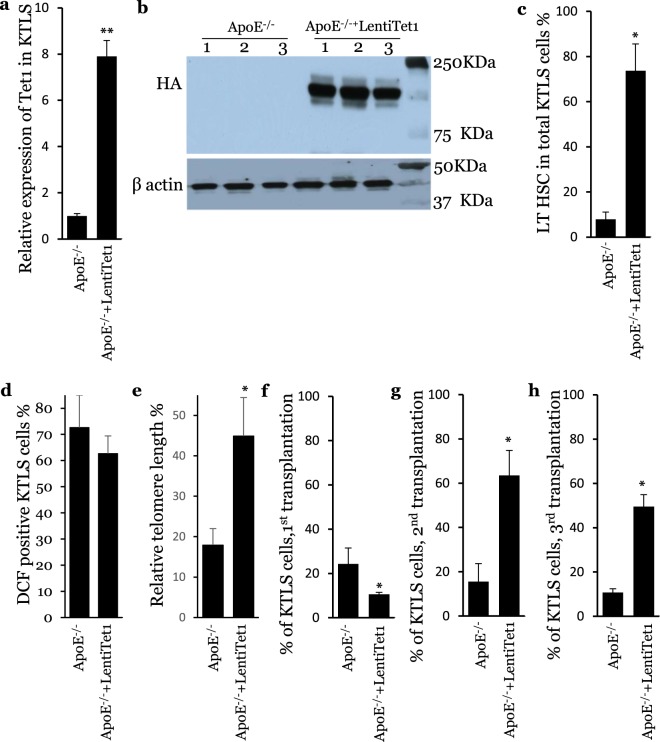
The expression of the Tet1 catalytic domain rescues quiescence, long-term population, life span and reconstitution capacity of HSCs isolated from ApoE^−/−^ mice. (**a)** Relative RNA expression of Tet1 catalytic domain. **(b)** Protein expression of Tet1 catalytic domain. (n = 4, **p < 0.01; vs ApoE^−/−^). (**c**) Long-term populations in ApoE^−/−^ and ApoE^−/−^+LentiTet1 HSCs. (**d**) Oxidant levels of ApoE^−/−^ and ApoE^−/−^+LentiTet1 HSCs. (**e**) Telomere length of ApoE^−/−^ and ApoE^−/−^+LentiTet1 HSCs. (**f**) Reconstitution capacity of ApoE^−/−^ and ApoE^−/−^+LentiTet1 HSCs after the first transplantation. (**g**) Reconstitution capacity of of ApoE^−/−^ and ApoE^−/−^+LentiTet1 HSCs after the second transplantation. (**h**) Reconstitution capacity of ApoE^−/−^ and ApoE^−/−^+LentiTet1 HSCs after the third transplantation. (n = 6, *p < 0.05; vs ApoE^−/−^).

We also expressed the Tet1 catalytic domain in HSCs isolated from ApoE^−/−^ mice (Fig. 5a,b). The LT population of HSCs was significantly larger than that of control HSCs from ApoE^−/−^ mice (Fig. 5c). The oxidant stress trended lower (Fig. 5d), but the telomere length was significantly restored (Fig. 5e). Similar to the results described in Fig. 4f, HSCs expressing the Tet1 catalytic domain displayed a limited reconstitution capacity after the first transplantation (Fig. 5f). However, these cells reconstituted 62.7% of HSCs in the recipient bone marrow after the second transplantation and 48.3% of HSCs after the third transplantation, which was significantly greater than the reconstitution capacity of the control HSCs from ApoE^−/−^ mice (Fig. 5g,h). To address the question why HSCs expressing the Tet1 catalytic domain manifest a limited reconstitution capacity after the first transplantation, we measured the proliferating and apoptotic populations in HSCs expressing the Tet1 catalytic domain. The expression of the Tet1 catalytic domain significantly reduced the proliferative Ki67^+^ populations in Tet1^−/−^ and ApoE^−/−^ HSCs before transplantation (Supplementary Figs. 5a and 6a). No significant difference was found in the Annexin V^+^ populations in the HSCs expressing the Tet1 catalytic domain (Supplementary Figs. 5b and 6b). These results indicate that the restoration of Tet1 activity rescues the reduced quiescence and LT populations of HSCs, and accordingly improves the reconstitution capacity of HSCs from ApoE^−/−^ mice after the second and third competitive transplantations.

### Tet1 deficiency upregulates p19 and p21 expression by decreasing H3K27me3 modifications in HSCs

**Figure 6:**
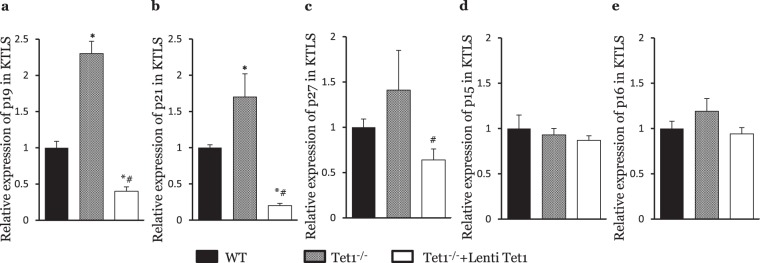
Expression levels of CIP/KIP and INK families in WT, Tet1^−/−^ and Tet1^−/−^+LentiTet1 HSCs. (**a)** Relative expression of p19 in KTLS. **(b)** Relative expression of p21 in KTLS. **(c)** Relative expression of p27 in KTLS. **(d)** Relative expression of p15 in KTLS. **(e)** Relative expression of p16 in KTLS. (n = 6, *p < 0.05; vs WT; ^#^p < 0.05; vs Tet1^−/−^).

In our previous study, we have shown that cell cycle regulators, including p19, p21 and p27, were upregulated and ultimately responsible for the decreased LT population and reconstitution capacity of HSCs from hypercholesterolemic mice^24^. Given this result, we screened the expression of the INK and KIP families in HSCs isolated from Tet1^−/−^ mice. Tet1^−/−^ HSCs showed significantly greater expression of p19 and p21 (Fig. 6a,b), but the expression of p15, p16 and p27 HSCs was the same as in WT mice (Fig. 6c,d,e). In addition, the expression of the Tet1 catalytic domain significantly reduced the expression of p19, p21 and p27 in HSCs isolated from Tet1^−/−^ mice (Fig. 6a–c).

**Figure 7:**
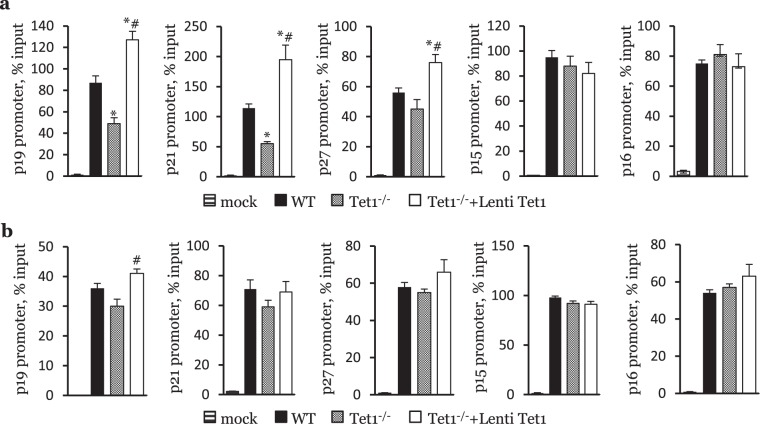
Tet1 regulates the H3K27me3 and H3K36me3 modifications on the chromatin of p19, p21 and p27. (**a)** H3K27me3 modification on the chromatin of p19, p21, p27, p15 and p16 in WT, Tet1^−/−^ and Tet1^−/−^+LentiTet1 HSCs. (n = 6, *p < 0.05; vs WT; ^#^p < 0.05; vs Tet1^−/−^). **(b)** H3K36me3 modification on the chromatin of p19, p21, p27, p15 and p16 in WT, Tet1^−/−^ and Tet1^−/−^+LentiTet1 HSCs. (n = 6, *p < 0.05; vs WT**;** ^#^p < 0.05; vs Tet1^−/−^).

Tet-dependent DNA demethylation typically increases the transcription of target genes^14,18^. Furthermore, one study has shown that Tet1 preferentially binds to CpG-rich sequences, facilitating the recruitment of PCR2 to CpG-rich promoters, and contributing to H3K27me3-mediated changes in gene repression^30^. In addition, Tet2 has been shown to promote H3K36me3-mediated gene activation^31,32^. To examine the molecular mechanisms underlying the effects of Tet1 deficiency on the expression of p19 and p21, we next sought to characterize the changes in histone modification and DNA methylation in the regulatory regions of our genes of interest. While pyrosequencing analysis did not show any significant difference in the methylation status of p19 and p21 promoters (Supplementary Fig. 7, 8), ChIP-PCR revealed that Tet1 deficiency significantly decreased the H3K27me3 modification in p19 and p21 (Fig. 7a) but did not affect the H3K36me3 modification in these two genes (Fig. 7b). The expression of the Tet1 catalytic domain significantly increased the H3K27me3 modification in p19, p21 and p27 (Fig. 7a), as well as the H3K36me3 modification in p19 (Fig. 7b). These results indicate that TET1 regulates the expression of the CIP/KIP and INK families by fine-tuning the bivalent H3K27me3 and H3K36me3 modifications on the chromatin of these genes.

## Discussion

Hypercholesterolemia, identified as a common risk factor of cardiovascular diseases and a co-morbidity of obesity, has been shown to increase the risk of all-cause mortality and morbidity^33–35^. Substantial investigations have linked hypercholesterolemia to a variety of deleterious alterations, including accelerated aging^36,37^, atherosclerosis and thrombosis^38^ and cancers^39^. It remains a critical challenge to understand the pathological mechanisms by which hypercholesterolemia imposes deleterious effects on such a wide range of tissues and organs. Here, we show that hypercholesterolemia decreased the expression of *Tet1* in HSCs and subsequently upregulated the expression p19 and p21 by reducing repressive H3K27me3. These changes caused loss of quiescence, telomere erosion, shortened life span and impaired reconstitution capacity of HSCs, leading to accelerated HSC aging phenotypes.

In humans, age-related hematopoietic changes include decreased bone marrow cellularity^40^, elevated incidence of thrombosis, increased myeloproliferative diseases^41^, late onset anemia^42^ and reduced regenerative potency and adaptive immunity^43,44^. Aging of the HSC compartment is thought to contribute to the occurrence of these clinical conditions^45^. Therefore, the elucidation of the mechanisms that cause aging of HSC could conceivably lead to strategies to prevent or even reverse the decline in immune function with age. Furthermore, manipulation of these mechanisms might allow the *ex vivo* expansion of HSCs without exhaustion. Our study showed that the deficiency of Tet1 resulted in a significant expansion of HSCs in mouse bone marrow. We also found that HSCs isolated from Tet1^−/−^ mice displayed a loss of quiescence and a reduction in life span due to a dramatic decrease in LT populations and quiescent side populations. The findings were supported by previous investigations made in Tet2^−/−18^ and Tet1^−/−^ mice^28^, and have been well established as hallmarks for HSC aging in murine models^11^. These results indicate that Tet1 is a critical regulator of the quiescence and the LT population of HSCs. We thus conclude that Tet1 deficiency causes a functional decline consistent with aging of the HSC compartment in the mouse.

A long-term goal in the studies of HSC aging is to develop strategies to repair or restore the functions that are impaired by aging processes. When we restored the activity of Tet1 by expressing the Tet1 catalytic domain in HSCs isolated from hypercholesterolemic or Tet1^−/−^ mice, the life span and reconstitution capacity of HSCs from Tet1^−/−^ mice significantly increased. The cells expressing the Tet1 catalytic domain had an improved long-term reconstitution capacity after the second and third transplantation. In HSCs isolated from hypercholesterolemic ApoE^−/−^ mice, the expression of the Tet1 catalytic domain also rescued their reconstitution capacity. Consistent with this increased reconstitution capacity, HSCs which expressed the Tet1 catalytic domain possessed significantly increased LT populations and quiescent side populations. These findings indicate that restoring Tet1 activity is a practical approach to recover the aging related exhaustion of the LT population and the decline in the reconstitution capacity of the HSC compartment in hypercholesterolemic mice.

The CIP/KIP and INK4 cell cycle inhibitor families participate in the maintenance of HSC quiescence, thereby governing the available size of the HSC compartment^46–48^. The increased expression of these cell cycle inhibitors has been observed in aged HSCs^29,49^. In the current study, the expression of p19 and p21 was upregulated in HSCs isolated from Tet1^−/−^ mice. This is consistent with our previous study, which showed paradoxically upregulated expression of p19 and p21 in HSCs from hypercholesterolemic mice^24^. Indeed, the inhibition of CIP/KIP or INK4 families rescued the quiescent LT population and the reconstitution capacity of aged HSCs^24,29^. The restoration of Tet1 activity synergistically decreased the expression of p19, p21 and p27 in HSCs from Tet1^−/−^ and ApoE^−/−^ mice, which led to the dramatic increase of the long-term and quiescent HSC populations. Another unexpected finding in our study is that the expression of the Tet1 catalytic domain limited the short-term reconstitution capacity of HSCs after the first HSC transplantation. This limitation of short-term reconstitution capacity of HSCs might be due to the inhibition of p19, p21 and p27, as well as to the dramatic increase in the quiescent long-term HSC population that possesses a very low proliferative activity.

Taken together, our results indicate that hypercholesterolemia downregulates the expression of Tet1 in HSCs which, in turn, increases the expression of p19 and p21. These molecular changes result in the exhaustion of long-term and side populations in the HSC compartment, leading to an acceleration of the HSC aging phenotype while the restoration of Tet1 activity reverses these deleterious effects. These findings provide a possibility to rescue the aging-related decline in HSCs. In addition, these findings might be helpful to understand the deleterious effects of hypercholesterolemia in a wide range of pathological processes.

## Experimental Procedures

### Mice

ApoE^−/−^, Tet1^+/−^, CD45.2 and CD45.1 WT mice were purchased from Jackson Laboratories (Bar Harbor, ME) and were maintained in a mouse barrier facility. Tet1^−/−^ mice were generated by inbreeding Tet1^+/−^ mice and their genotyping was confirmed by PCR. Care of mice was in accordance with NIH guidelines, and the Institutional Animal Care and Use Committee of the University of Massachusetts Medical School approved all protocols. Mice were kept on a 12 hr light/dark schedule and were allowed free access to chow and water. ApoE^−/−^, Tet1^+/−^, Tet1^−/−^ and WT mice were fed standard mouse chow (5.4 g fat/100 g diet, 0.01% cholesterol). Two different hypercholesterolemic mouse models were applied in this study. One was ApoE^−/−^ mouse fed standard mouse chow. Another was the diet induced hypercholesterolemic mice (HCD mice) which were WT mice fed a hypercholesterolemic diet with 10 g fat/100 g diet, 11.25 g cholesterol/100 g diet (Research Diets, New Brunswick, NJ). These two mouse models have been extensively used as standard hypercholesterolemic models in different labs.

### Flow cytometry and HSC isolation

Cells were stained with monoclonal antibodies conjugated to various fluoroprobes. These antibodies included: cKit (2B8), Sca-1 (E13–161.7), CD150, CD48, CD90.1, CD34, Flk2, CD45.1, CD45.2 and the lineage cocktail consisted of CD4, CD8, B220 (RA3-6B2), TER-119, Mac-1 (MI/70). All antibodies were purchased from BD Bioscience (San Diego, CA). FACS analysis was carried out on a FACSAria or MoFlow. HSCs were isolated from the bone marrow and defined as cKit^+^ sca-1^+^ CD90.1^lo/−^Lin^−^. Long-term HSCs (LT-HSCs), phenotypically defined as CD34^−^ Flk_2_^−^KTLS cells. Short-term HSCs, phenotypically defined as CD34^+^Flk_2_^−^KTLS cells^24^.

### *In vitro* culture of HSCs

KTLS cells were cultured in Minimum Essential Media, alpha modification (αMEM Sigma) containing 12.5% FCS (JRH Bioscience), 12.5% horse serum (Gibco BRL) and 1 nM dexamethasone^24^.

### Lentiviral particle preparation and transduction

The Tet1 specific and control shRNA plasmids were purchased from Santa Cruz (CA, USA). The plasmid with Tet1 catalytic domain (pTYF-U6-shCONT-EF1-Puro-2A-CD1) was a gift from Dr. Yi Zhang (Boston Children’s Hospital, Boston, MA). The envelope and helper plasmids were purchased from ABM (Toronto, Canada). The lentiviral particles were prepared according to the kit instructions. Freshly isolated KTSL cells were transduced with lentivirus for 24 hours and then selected with puromycin (2 μg/ml) (Santa Cruz Biotechnology, CA, USA) for 72 hours^17^.

### Competitive bone marrow reconstitution assay

We sorted KTLS cells or LT-HSC from three-month old CD45.2 WT, CD45.1 WT or Tet1^−/−^ mice (CD45.2). The recipient mice were three-month old CD45.1 congenic mice. Each recipient mouse was lethally irradiated and transplanted with 3000 KTLS cells or LT-HSC cells by retro-orbital injection. Among the transplanted KTLS or LT-HSCs, 1500 cells were support cells isolated from CD45.1 WT. The other 1500 cells were isolated from CD45.2 WT or Tet1^−/−^ mice (CD45.2)^24^. The reconstitution of HSC compartment in recipient mice was analyzed 3 months after transplantation. Six recipient and six donor mice were included in this analysis.

### RT–PCR

RNA was isolated from cells using RNAqueous-Micro kit (Ambion Life Technologies). Transcription to cDNA was performed using SuperScript III (Invitrogen). The primers were purchased from IDT. All PCRs were carried out in triplicate using an Eppendorf Mastercycler (Eppendorf). All genes are normalized to 18 s rRNA. Primer sequences are shown in Supplementary Table 1.

### Analysis of oxidant stress

We loaded samples of cultures with DCF‐DA (Sigma) and incubated them on a shaker at 37 °C for 30 min. The peak excitation wavelength for oxidized DCF was 488 nm, and emission was 525 nm^24^. The DCF positive cells were analyzed with FACS.

### Telomere length measurement

Telomere length was measured by telomere PNA kit/FITC (DAKO)^24^. In brief, the sample DNA was denatured for 10 minutes at 82 °C in a microcentrifuge tube either in the presence of hybridization solution without probe or in hybridization solution containing fluorescein‐conjugated PNA telomere probe. Then hybridization took place in the dark at room temperature (RT) overnight. The hybridization was followed by two 10‐minute post‐hybridization washes with a Wash Solution at 40 °C. The sample was then resuspended in an appropriate buffer for further flow cytometric analysis. DNA Staining Solution included in the kit was used for identification of G0/G1 cells. After flow cytometric analysis, the data obtained were used for determination of a relative telomere length (RTL). The RTL value was calculated as the ratio between the telomere signal of each sample and the control cells with correction for the DNA index of G0/G1 cells.

### Chromatin Immunoprecipitation (ChIP)

ChIP was performed as described previously^50^. Approximately 1 × 10^6^ HSCs were incubated for 10 min at room temperature with 1% formaldehyde. After cross-linking, the reaction was quenched with 0.25 M glycine for 10 min at room temperature. Proteins were initially cross-linked to DNA and nuclei were then pelleted and sonicated to 200–500 bp fragments (Bioruptor, Diagenode). The cross-linked DNA was immunoprecipitated with H3K27me3 antibody (Millipore, USA) overnight at 4 °C with rotation. DNA-Antibody complexes were bound to ChIP beads, pulled down, washed and then eluted from beads. Following reversal of cross-linkage, purified DNA was used for Quantitative PCR using ChIP PCR primers that were purchased from IDT (MA, USA). Immunoprecipitation efficiency was calculated by normalizing sample *C*_T_ values against control IgG values and calculating ratios of sample *C*_T_ values relative to input values.

### DNA extraction, bisulfite conversion and pyrosequencing

Pyrosequencing was performed as described previously^50^. Genomic DNA was extracted from HSCs using standard phenol/chloroform extraction followed by isopropanol precipitation and ethanol wash and quantified using a NanoDrop Spectrophotometer. 500 ng of DNA was used in the bisulfite conversion reactions where unmethylated cytosines were converted to uracil with the EZ DNA Methylation Gold^TM^ kit (Zymo Research) according to the manufacturer’s instructions. Briefly, DNA was mixed with CT conversion reagents and the conversion was run in a thermocycler (Biometra, Goettingen, Germany) at the recommended cycle conditions. Converted DNA was purified on a spin column and eluted into a total of 10 μl Buffer EB. PCR and pyrosequencing primer sets with one biotin-labelled primer were used to amplify the bisulfite converted DNA. New primers for each gene were designed using PyroMark Assay Design software version 2.0.1.15 (Qiagen). The size of the amplicons was around 100–200 bp.

PCRs were performed using a converted DNA by 2xHiFi Hotstart Uracil+Ready Mix PCR kit (Kapa Biosystems). Briefly, 5 μl master mix, 5 pmol of each primer, 20 ng genomic DNA and ultra-pure water to a final volume of 10 µl were mixed for each reaction and run at thermal cycling conditions: 95 °C for 3 min and then 50 cycles: 20 sec at 98 °C; 15 sec at the optimized primer-specific annealing temperature; 15 sec at 72 °C and a final extension for 1 min at 72 °C. The amplified DNA was confirmed by electrophoresis in a 2% agarose gel. 2 μl streptavidin beads (GE Healthcare, Buckinghamshire, UK), 40 μl PyroMark binding buffer, 10 μl PCR product and 28 μl water were mixed and incubated for 10 min on a shaking table at 1300 rpm. Using the Biotage Q96 Vaccum Workstation, amplicons were separated, denatured, washed and added to 25 μl annealing buffer containing 0.33 μM of pyrosequencing primer. Primer annealing was performed by incubating the samples at 80 °C for 2 min and allowing them to cool to room temperature prior to pyrosequencing. PyroGold reagents were used for the pyrosequencing reaction and the signal was analyzed using the PSQ 96MA system (Biotage, Uppsala, Sweden). Target CGs were evaluated by instrument software (PSQ96MA 2.1) which converts the pyrograms to numerical values for peak heights and calculates percentage of methylation at each base as a C/T ratio. Supplementary Fig. 8 is the schematic of the *p19* and *p21* genes showing the sequence and location of the primers and the CpG islands tested by pyrosequencing.

### Statistical analysis

All data were shown as means ± sd. Statistical analyses were carried out with either GraphPad Prism (GraphPad Software). Statistical significance was evaluated by using a one- or two-way analysis of variance (ANOVA) or an unpaired t-test. Significance was established for *P* values <0.05. Adjustment for multiplicity of comparisons was not utilized.

## Supplementary information

Supplementary information.
