# Supplementary material for: Hypercholesterolemia Accelerates the Aging Phenotypes of Hematopoietic Stem Cells by a Tet1-Dependent Pathway
Source: Sci Rep. 2020 Feb 27;10:3567. doi: 10.1038/s41598-020-60403-w (PMC7046636; doi:10.1038/s41598-020-60403-w)
Supplement: Supplementary file 1 — Supplementary information. [file 41598_2020_60403_MOESM1_ESM.pdf]

Hypercholesterolemia Accelerates the Aging Phenotypes of Hematopoietic Stem Cells by a Tet1-Dependent Pathway

Guodong Tie[1*], Jinglian Yan[1*], Lyne Khair[1], Amanda Tutto[1], Louis M. Messina[1,2]

Division of Vascular and Endovascular Surgery, University of Massachusetts Medical School, Worcester, MA 01655, USA.

Correspondence: Louis M. Messina, MD

E-mail: Louis.Messina@umassmemorial.org

[*] The authors contribute to the manuscript equally.

Running Title: Tet1 deficiency accelerates HSC aging phenotypes

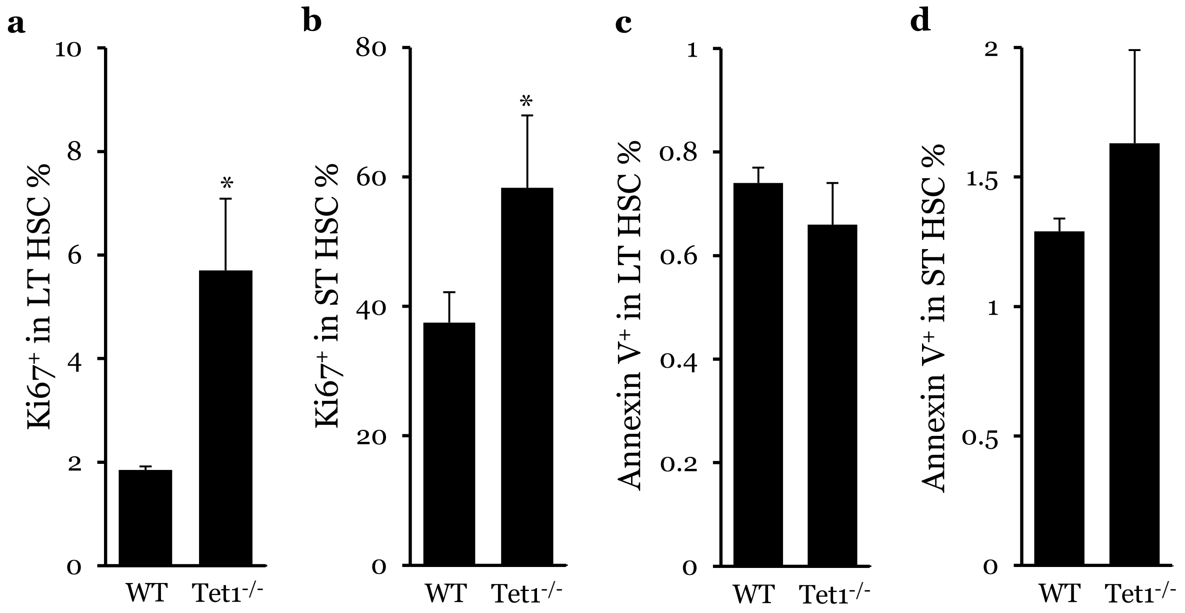

Supplementary Figure 1. The proliferative and apoptotic populations in long-term and short term HSCs isolated from WT and Tet1$^{-/-}$ mice. **(a)** Ki67^{+} cells in LT HSCs of WT and Tet1$^{-/-}$ mice. **(b)** Ki67^{+} cells in ST HSCs of WT and Tet1$^{-/-}$ mice. **(c)** Annexin V^{+} cells in LT HSCs of WT and Tet1$^{-/-}$ mice. **(d)** Ki67^{+} cells in ST HSCs of WT and Tet1$^{-/-}$ mice. (n=6, *, p<0.05, vs WT).

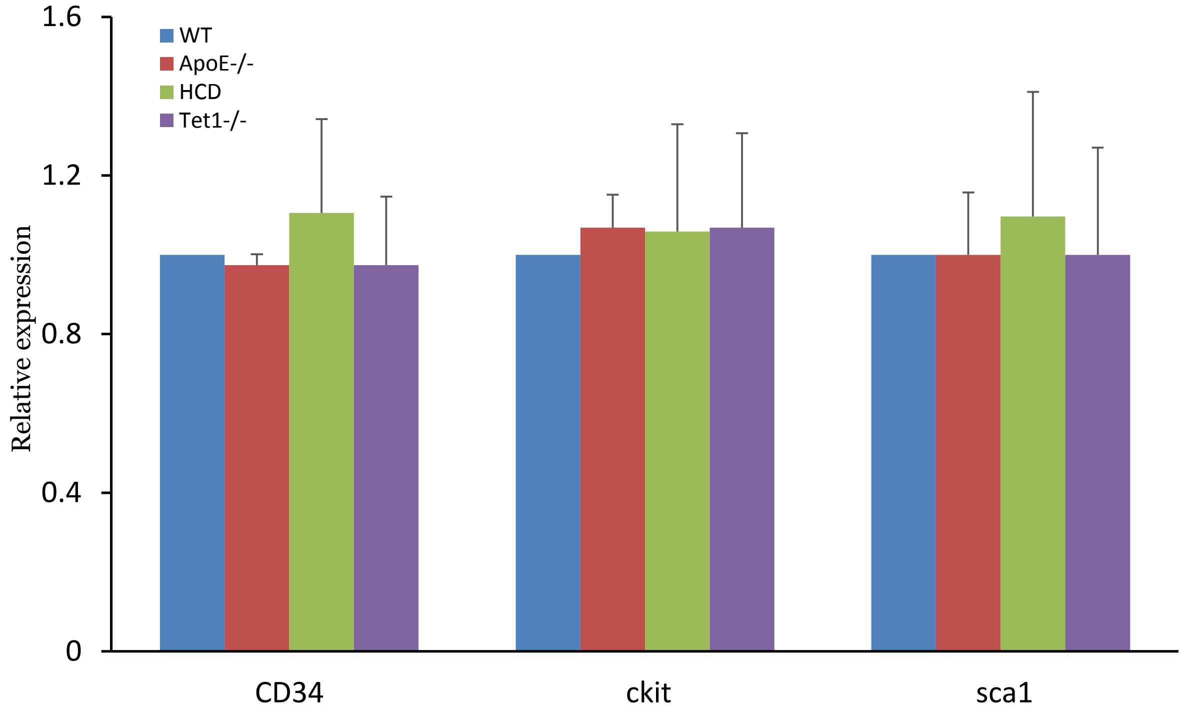

Supplementary Figure 2. Hypercholesteromia and Tet1 deficiency don't change the expression of HSC surface markers. (n=6, vs WT).

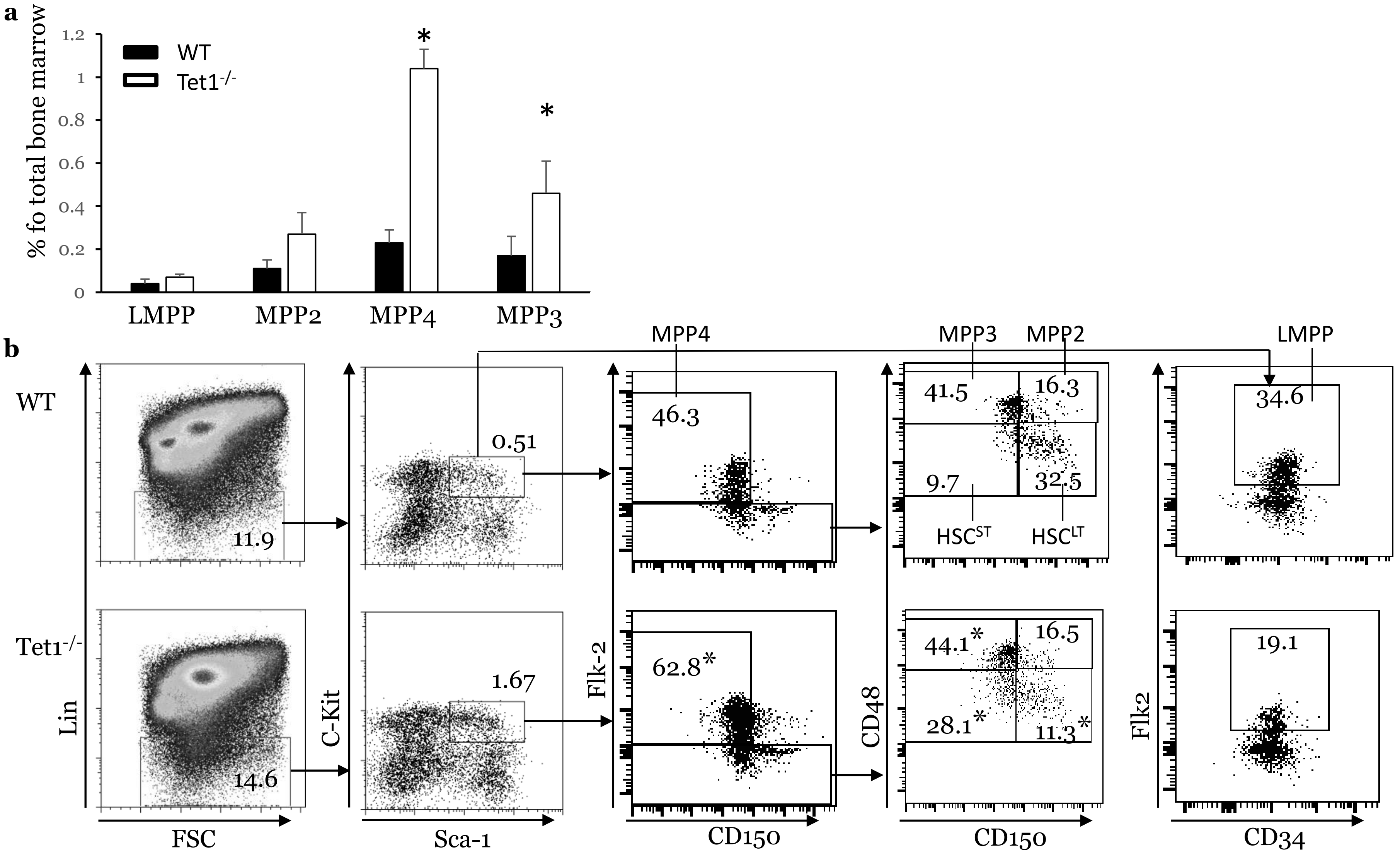

Supplementary Figure 3. Tet1 deficiency increases short term HSC, MMP3 and MPP4 compartments, and decreases long term HSC compartment. (a) The ratio of LMPP, MPP2, MPP3 and MPP4 in total bone marrow (n=4, *, $p<0.05$; vs WT). **(b)** The representative FACS analysis of LMPP, MPP2, MPP3 and MPP4. Long term HSCs were identified as Lin^- $Sca\text{-}1^+$ $cKit^+$ $Flk2^-$ $CD150^+$ $CD48^-$. Short term HSCs were identified as Lin^- $Sca\text{-}1^+$ $cKit^+$ $Flk2^-$ $CD150^-$ $CD48^-$. LMPP was identified as Lin^- $Sca\text{-}1^+$ $cKit^+$ $Flk2^{hi}$ $CD150^-$ $CD34^+$. MPP2 was identified as Lin^- $Sca\text{-}1^+$ $cKit^+$ $Flk2^-$ $CD150^+$ $CD48^+$. MPP3 was identified as Lin^- $Sca\text{-}1^+$ $cKit^+$ $Flk2^-$ $CD150^-$ $CD48^+$. MPP4 was identified as Lin^- $Sca\text{-}1^+$ $cKit^+$ $Flk2^+$ $CD150^-$.

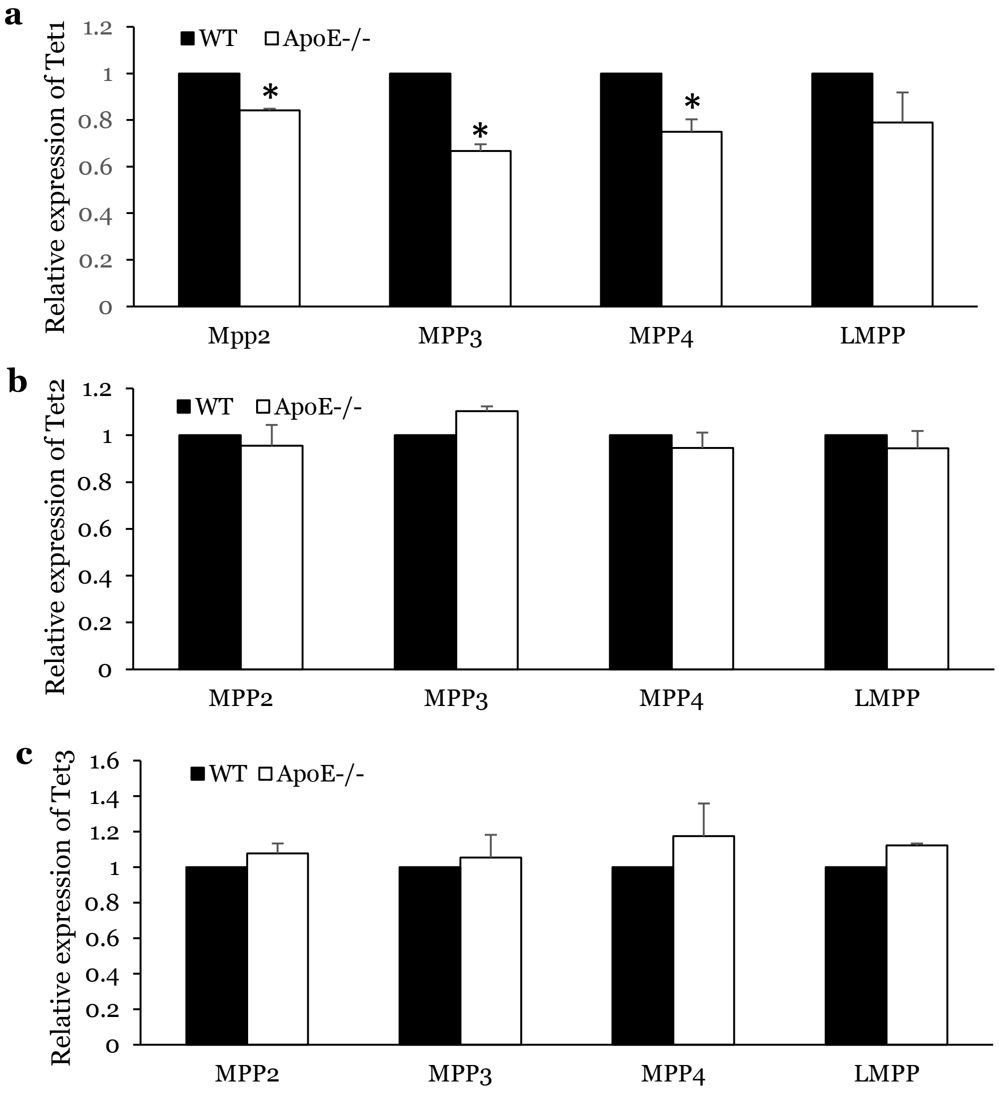

Supplementary Figure 4. The expression of Tet1, Tet2 and Tet3 in hematopoitic progenitor cells, including MPP2, MPP3, MPP4 and LMPP. **(a)** The expression Tet1 in MPP2, MPP3, MPP4 and LMPP isolated from WT and ApoE$^{-/-}$ mice. **(b)** The expression Tet2 in MPP2, MPP3, MPP4 and LMPP isolated from WT and ApoE$^{-/-}$ mice. **(c)** The expression Tet3 in MPP2, MPP3, MPP4 and LMPP isolated from WT and ApoE$^{-/-}$ mice. (n=4, *, $p<0.05$, vs WT).

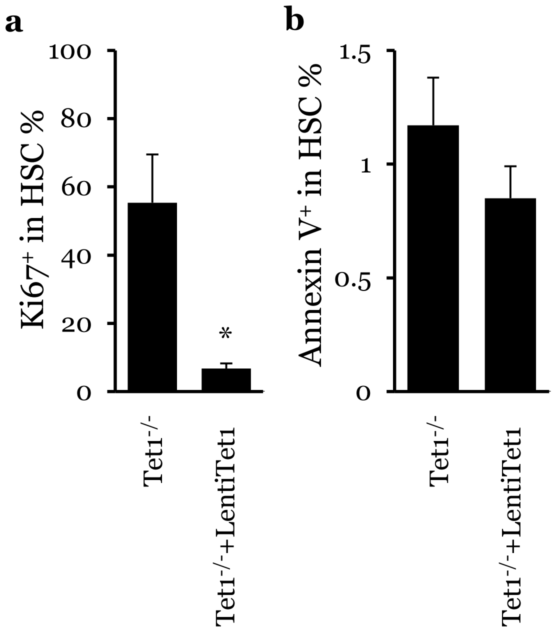

Supplementary Figure 5. The proliferative and apoptotic populations in control Tet1$^{-/-}$ HSCs and Tet1$^{-/-}$ HSCs expressing Tet1 catalytic domain. (a) Ki67^{+} cells in control Tet1$^{-/-}$ HSCs and Tet1$^{-/-}$ HSCs expressing Tet1 catalytic domain. **(b)** Annexin V^{+} cells in control Tet1$^{-/-}$ HSCs and Tet1$^{-/-}$ HSCs expressing Tet1 catalytic domain. (n=6, *, $p<0.05$, vs Tet1$^{-/-}$).

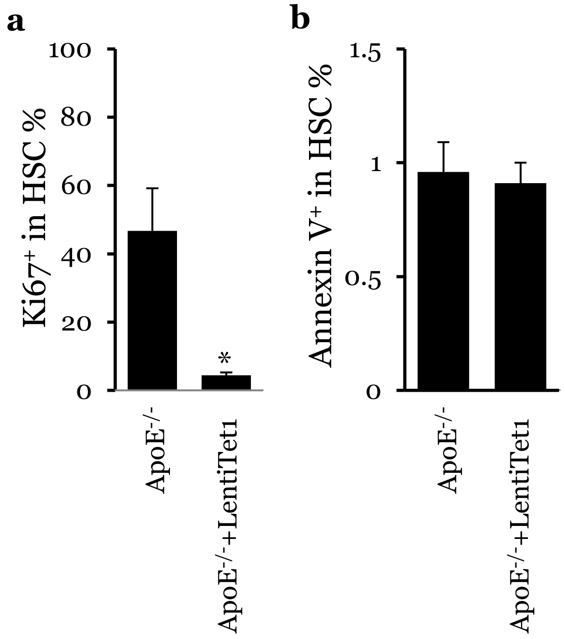

Supplementary Figure 6. The proliferative and apoptotic populations in control ApoE$^{-/-}$ HSCs and ApoE$^{-/-}$ HSCs expressing Tet1 catalytic domain. (a) Ki67^{+} cells in control ApoE$^{-/-}$ HSCs and ApoE$^{-/-}$ HSCs expressing Tet1 catalytic domain. **(b)** Annexin V^{+} cells in control ApoE$^{-/-}$ HSCs and ApoE$^{-/-}$ HSCs expressing Tet1 catalytic domain. (n=6, *, $p<0.05$, vs ApoE$^{-/-}$).

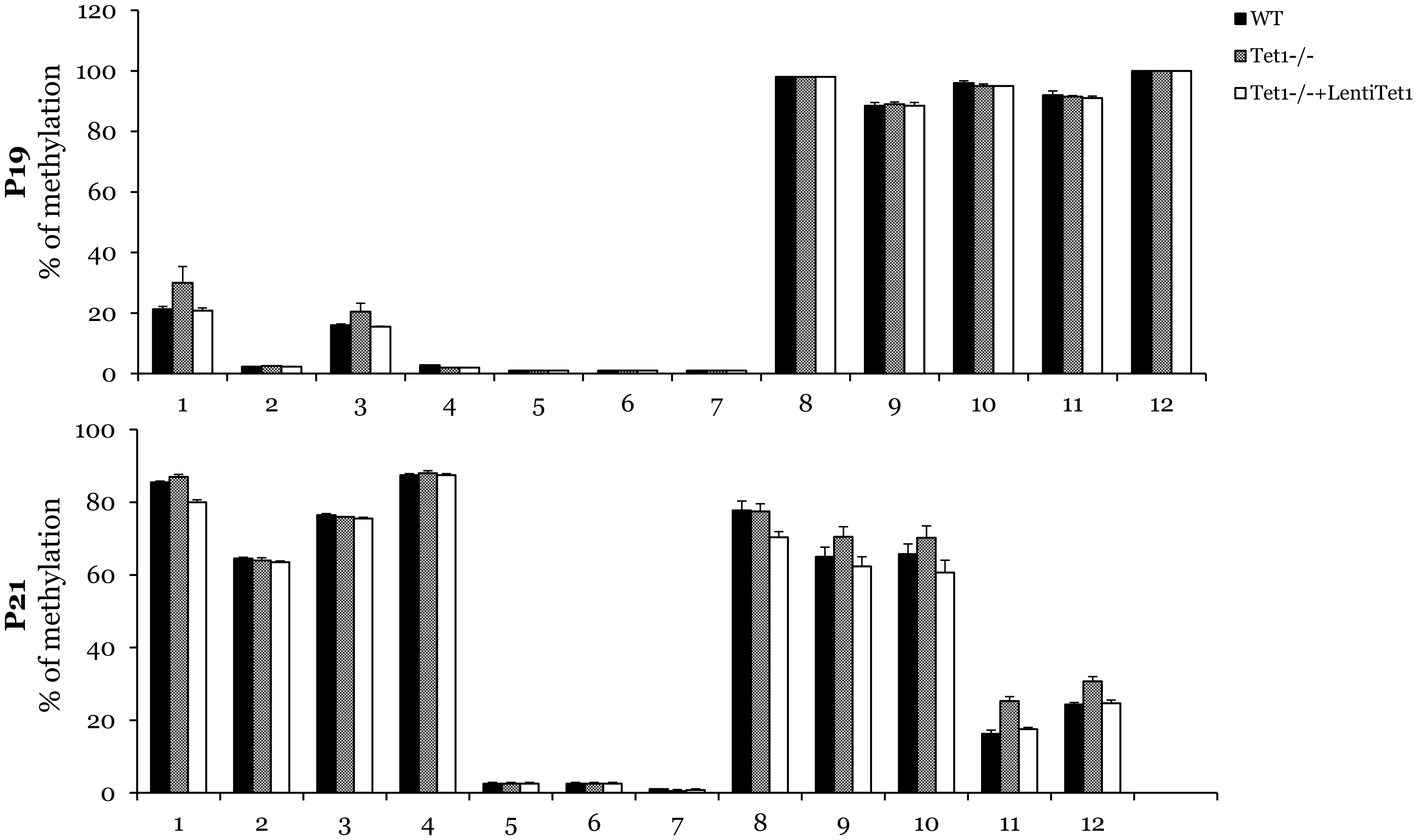

Supplementary Figure 7. DNA methylation status of the promoters of p19 and p21 in WT, Tet1$^{-/-}$ and Tet1$^{-/-}$+LentiTet1 HSCs. (n=6, *, p<0.05; vs WT; #, p<0.05; vs Tet1$^{-/-}$)..

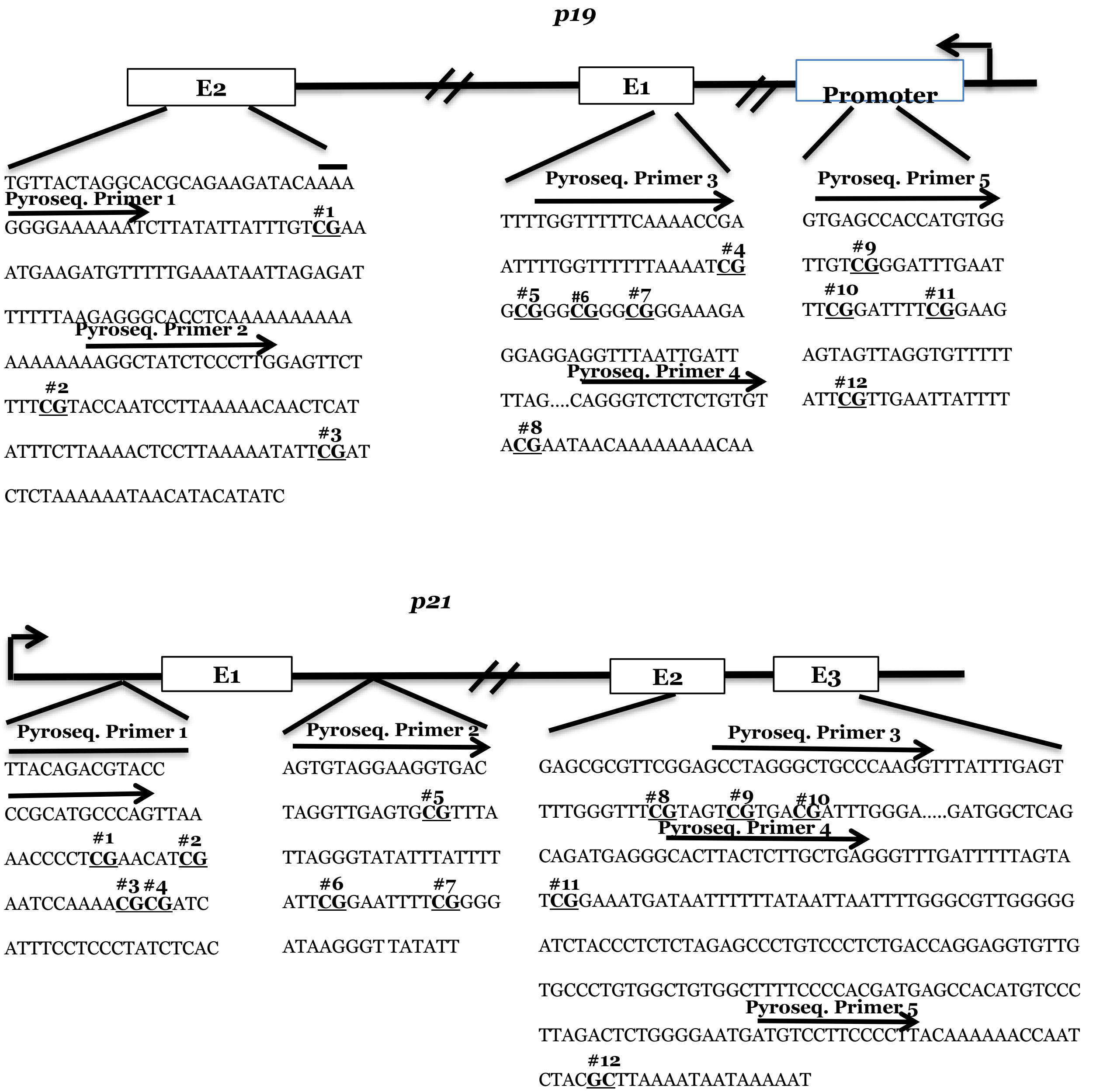

Supplementary Figure 8. Schematic of the *p19* and *p21* genes showing the sequence and location of the primers and the CpG islands tested by pyrosequencing

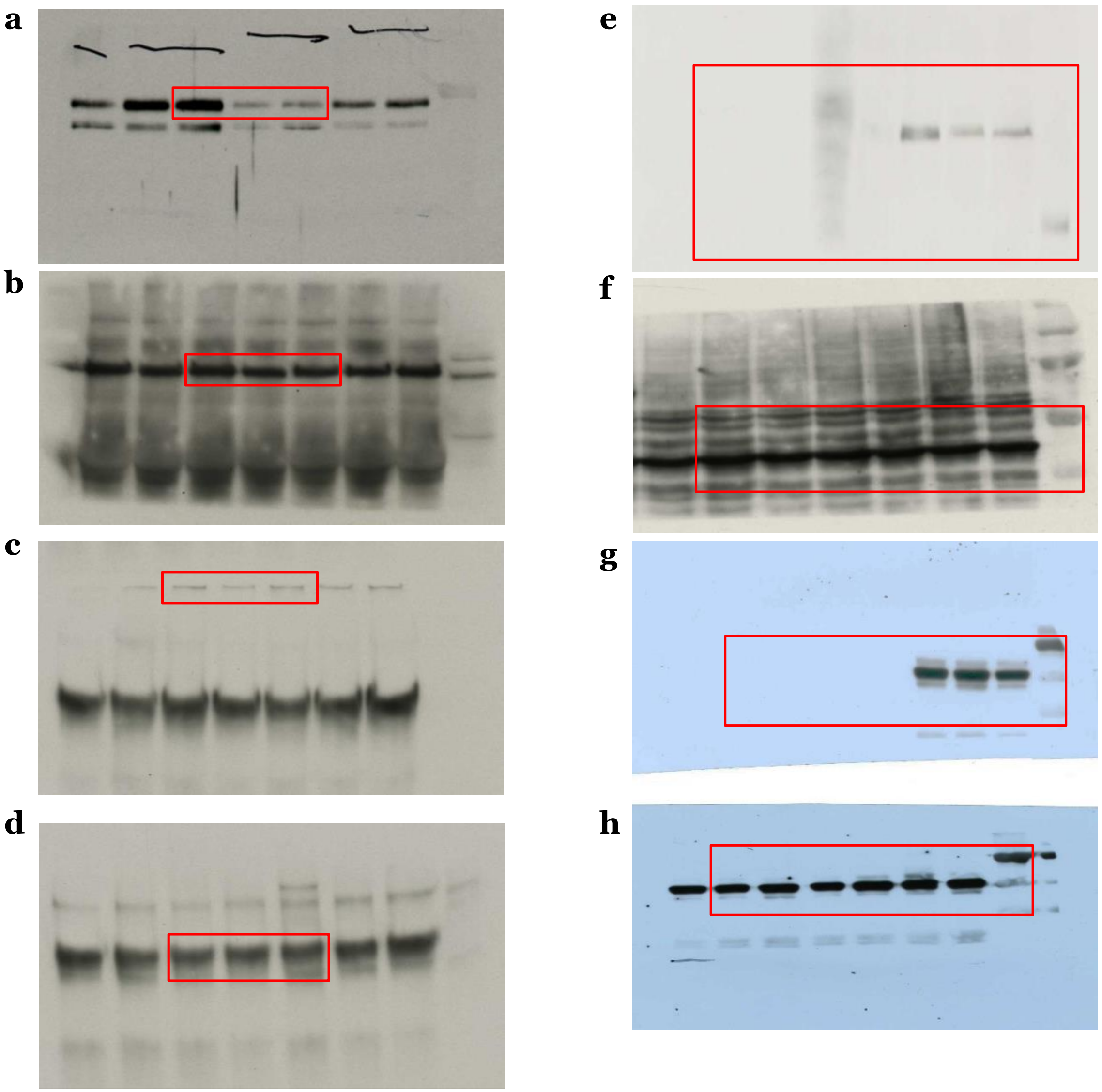

Supplementary Figure 9. Uncroped western blot images. (a) Figure 1d first panel uncropped western blot image. **(b)** Figure 1d second panel uncropped western blot image. **(c)** Figure 1d third panel uncropped western blot image. **(d)** Figure 1d fourth panel uncropped western blot image. **(e)** Figure 4a first panel uncropped western blot image. **(f)** Figure 4a second panel uncropped western blot image. **(g)** Figure 5b first panel uncropped western blot image. **(h)** Figure 5b second panel uncropped western blot image. The red box areas show the lanes cropped for the final figures.

Genes	Forward primer	Reverse primer
18s	CGGCTACCACATCCAAGGAA	GCTGGAATTACCGCGGCT
P15	GGTGGGTGCAGTCAGTACCT	CGAGCTGGAGGTGACTTCTC
P16	CAACGCCCCGAACTCTTTC	GCAGAAGAGCTGCTACGTGAAC
P19	CGGTATCCACTATGCTTCTGGAA	CCGCTGCGCCACTCAA
P21	TTCCGCACAGGAGCAAAGT	CGGCGCAACTGCTCACT
P27	GGCCCGGTCAATCATGAA	TTGCGCTGACTCG CTTCTTC
Tet1	CAGGAGAGGGACATAAAGACTGATA	AGAGAGAGAGACATTTCAAGTGCAT
Tet2	TGTTGTTGTCAGGGTGAGAATC	TCTTGCTTCTGGCAAACTTACA
Tet3	CCGGATTGAGAAGGTCATCTAC	AAGATAACAATCACGGCGTTCT
CD34	ACCACAGACTTCCCCAACTG	CGGATTCCAGAGCATTTGAT
C-KIT	TCATCGAGTGTGATGGGAAA	CGCACTTTGAGGGTGAATTT
Sca-1	CTGATGGTCCTCCCAATGAC	GAGCACCTACCTACCCAGCA

Supplementary Table 1. Mouse primers used for quantitative real time PCR
